# Cardiac Threat Appraisal and Depression after First Myocardial Infarction

**DOI:** 10.3389/fpsyg.2012.00365

**Published:** 2012-10-08

**Authors:** Claus Vögele, Oliver Christ, Heike Spaderna

**Affiliations:** ^1^Integrative Research Unit on Social and Individual Development, University of LuxembourgWalferdange, Luxembourg; ^2^Department of Psychology, University of MarburgMarburg, Germany; ^3^Department of Psychology, University of MainzMainz, Germany

**Keywords:** myocardial infarction, depression, cardiac threat appraisal, illness adaptation

## Abstract

The present study investigated cardiac threat appraisal and its association with depression after first myocardial infarction (MI). A semi-structured interview allowing for DSM-IV-axis I diagnoses was administered to 36 patients after first MI. Patients completed self-reports 5–15 days after the MI (time 1), 6–8 weeks later (time 2), and again 6 months later (time 3). Assessments at time 1 included indices of cardiac threat appraisal, locus of control, coping, and depression while at time 2 and time 3 only measures of depression were obtained. Cardiac threat appraisal was significantly correlated with depression at time 1, but was unrelated to depression scores at time 2 and time 3. Furthermore, there was a significant inverse association between cardiac threat appraisal and the subscales “search for affiliation” and “threat minimization” of the coping questionnaire. Additionally, “search for affiliation” correlated negatively with depression scores at time 1 and time 3, and “threat minimization” negatively with depression scores at time 1 and time 2. These results suggest a significant association between cardiac threat appraisal and depressive symptoms shortly after MI. Practical implications for treatment are discussed.

## Introduction

Previous research on changes in psychological well-being and mental disorders following myocardial infarction (MI) has mostly focused on the role of depression in disease progression. Several studies have documented a high prevalence of depression in patients recovering from an MI (Rozanski et al., 2005; Thombs et al., 2006; Celano and Huffman, 2011). In women, post-MI depression is significantly more prevalent than in men (Naqvi et al., 2005). There is evidence that post-MI depression is an independent risk factor for cardiac events and mortality (Nicholson et al., 2006; Frasure-Smith and Lespérance, 2008), hospital readmissions (Dodson et al., 2012), and relates to adverse quality of life outcomes (Myers et al., 2012). In contrast to this evidence on the consequences of post-MI depression, little is known about factors contributing to the onset of post-MI depression. The present study aimed to investigate the role of cognitive factors in post-MI depression using a longitudinal design.

Medical factors appear to contribute at best marginally to the presence (Doyle et al., 2011) and onset of post-MI depression (Spijkerman et al., 2005). Other risk factors that have been identified are female sex, life-events (Doyle et al., 2011), living alone (Spijkerman et al., 2005), depression before the MI (Spijkerman et al., 2005), and vital exhaustion (Spijkerman et al., 2005). As yet, however, little research has considered the contribution of cognitive factors such as threat appraisal, locus of control, or coping, to the development of depression in post-MI patients. The present study addresses these shortfalls using the stress-coping model of Lazarus and Folkman (1984) as the theoretical basis. The model posits that three mediating processes determine adjustment to illness: cognitive appraisal (including threat appraisal), coping strategies, and coping resources (including control beliefs).

Several studies have shown that patients are aware of several different kinds of threats following the infarction, for example uncomfortable physical sensations and complications, medical therapy and self-care, limitations to work/physical activity, social/interpersonal/family problems, and financial difficulties (Bennett, 1992). Perceived threat is associated with distress and depressive symptoms (Connell et al., 1994; Pakenham, 1999), and affects patients’ choice of coping strategies (Lazarus and Folkman, 1984). In turn, different forms of coping strategies may have differential effects on illness adaptation (Carver and Scheier, 1994). Patients who use emotion-focused strategies, e.g., rumination, tend to have more problems adapting to chronic illness than do those who use problem-focused strategies (Maes et al., 1996). Rumination has been defined as a mode of responding to distress that involves passively focusing one’s attention on symptoms of distress without taking action (Nolen-Hoeksema, 1991). This dysfunctional response style has been shown to intensify depressed mood, to impair interpersonal problem solving, and to lead to more pessimistic future perspectives and less social support. Similar results have been reported for patients with coronary heart disease showing that rumination is associated with emotional distress after cardiac surgery (Schröder et al., 1998), and with perceived stress in patients scheduled for angiography (Closa Leon et al., 2010). In patients after an MI rumination was positively associated with depression (Garnefski et al., 2009) and predicted depression scores 1 year later (Garnefski and Kraaji, 2010). Rumination may, therefore, mediate the effects of perceived threat on depression, and thus show a positive association with perceived threat.

Another psychological factor impacting emotional adaption to illness and health outcomes is Locus of Control (Stanton et al., 2007). Health-related control beliefs result from knowledge about the controllability of illness and health and from attempts to influence physical processes. Internal control beliefs may increase subjective feelings of controllability of stressful situations (e.g., chronic illnesses) and, therefore, contribute to chronic illnesses to be experienced as less exhausting and stress inducing. Control beliefs can, therefore, affect primary threat appraisal in that persons with internal control beliefs appraise stressors as less threatening than persons with external control beliefs since they assess their resources as sufficient to cope with the stressful situation (Cordes, 1998). Several studies show a relation between fatalistic externality and depressive tendencies in patients with chronic illnesses (Stanton et al., 2007).

In summary, patients who feel threatened by their illness have a higher risk of developing depressive symptoms than patients who feel less threatened by their illness. Control beliefs are an important personality variable in illness adaptation: Patients with external fatalistic beliefs have a higher risk to develop depressive symptoms, whereas patients with internal control beliefs are more likely to adapt to chronic disease. Finally, threat appraisal can affect the choice of coping strategies.

The present study aimed at elucidating the unique contribution of cognitive factors to depression in the immediate and follow-up period of time following the MI. Such results would have potential implications for the design of interventions in this crucial period of rehabilitation. We hypothesize that post-MI patients with high threat appraisal are more likely to use ruminative coping strategies than patients who feel less threatened by the MI, and that this rumination in turn is associated with higher depression scores. Specifically, this study examines the following hypotheses: (1) Higher threat appraisal in post-MI patients is related to higher depression scores and a higher probability of a DSM-IV depression diagnosis. (2) Higher internality in post-MI patients is related to lower depression scores, while fatalistic externality relates to higher depression scores. (3) High threat appraisal is related to higher “rumination” scores. (4) Rumination is related to higher depression scores.

## Materials and Methods

### Study design and selection of participants

Data were collected at study recruitment (time 1) 5–15 days after the MI, 6–8 weeks later (time 2), and again 6 months later (time 3). At time 1, threat appraisal, control beliefs, coping, and depression were assessed. In addition, clinical and patient demographic data were obtained from the medical record and a demographic questionnaire, respectively. At time 2 and time 3, depression was re-assessed and information on disease progression/recovery was obtained from patients. At time 1, patients were contacted at the hospital, at time 2 and time 3, all patients had returned home.

Prior to enrollment in the study, written informed consent was obtained from all participants. Before commencement of the study, the study protocol was approved by the Hospital Ethics Committee. All participants met the following criteria: (a) first MI, (b) no other acute life threatening illness, (c) fluent in the assessment language (German), and (d) sufficient cognitive ability to respond to the questionnaires. 64.4% of the patients who met these criteria consented to participate in the study. The final sample consisted of 36 patients (32 men, 4 women). The mean age of the sample was 57.6 years (SD = 9.99), ranging from 39 to 77 years, 89% lived together with a partner. At time 2, 33 patients of the original sample could be contacted (91.7%) and at time 3, 24 patients (66.7%).

### Measures

#### Clinical and demographic data

At time 1, participants completed a questionnaire assessing patients’ age, educational level, profession, marital status, and number of children (if any). The following clinical variables obtained at time 1 were included in the study: blood lipids (total serum cholesterol, high-density-lipoprotein, and low-density-lipoprotein) and angiographic results (number of main coronary arteries with >70% stenosis, maximum stenosis, etc.). The rationale for including these indicators of severity of illness was to control for their effects on the focal variables of interest. At time 2 and time 3, a short telephone interview was conducted to enquire about the recovery process (e.g., medical complications, re-infarction, and length of stay in hospital).

#### Threat appraisal

For the assessment of threat appraisal, a German version of the Cardiac Event Threat Questionnaire (CTQ) was administered (Bennett et al., 1996). Two items that were irrelevant for patients treated within the German health care system were excluded (both items refer to threat posed by the cost of treatment). The remaining 29 items assess the degree of perceived threat in five different categories (Fatigue: e.g., “Needing more rest than usual”; General health: e.g., “My condition in general”; Disease-specific symptoms: e.g., “Tingling in my chest”; Work: e.g., “Returning to a stressful job”; Family: e.g., “The effects of my condition on my children”). Participants rated perceived threat on a four-point response scale (endpoints: 1 = not at all concerned, 4 = very concerned). Higher values indicate higher perceived threat. Cronbach’s alpha in the present study was 0.91 for the overall scale. Cronbach’s alpha for the CTQ-subscales ranged between 0.79 and 0.82.

#### Depression

A German version of the Center for Epidemiological Studies Depression Scale (CES-D; Radloff, 1977) was used to measure depressive symptoms (Allgemeine Depressionskala, ADS; Hautzinger and Bailer, 1993). The ADS consists of 20 items assessing on four-point response scales (0 = rarely or none of the time to 3 = most or all of the time) how often, during the past week, participants have felt or behaved in specific ways. Higher scores indicate higher levels of depressive symptoms. Cronbach’s alpha for the ADS obtained in the present sample was 0.76 (time 1), 0.78 (time 2), and 0.83 (time 3).

In addition to the psychometric assessment of depression using a standardized questionnaire, clinical diagnoses were established by using a structured clinical interview (“Diagnostisches Interview für Psychische Störungen – Forschungsversion,” F-DIPS, Diagnostic Interview for Mental Disorders – Research Version; Margraf et al., 1996). The F-DIPS is a well standardized, validated, and structured interview allowing the assessment of mental disorders on Axis I of the DSM-IV (American Psychiatric Association, 1994) and is based on the Anxiety Disorders Interview Schedule (ADIS-IV-L; DiNardo et al., 1995). In addition to current diagnoses, this interview also permits the retrospective recording of depressive episodes prior to MI. F-DIPS interviews were conducted by a trained clinical psychologist. Written interview transcripts were scored and diagnoses awarded by another trained psychologist who was blind to interviewees’ medical condition.

#### Locus of control

For the assessment of health-related locus of control, a German questionnaire (“Fragebogen zur Erhebung von Kontrollüberzeugungen zu Krankheit und Gesundheit,” KKG) was used (Lohaus and Schmitt, 1989). The KKG is based on the Multidimensional Health Locus of Control Scale (MHLC; Wallston et al., 1978), and consists of three subscales: internality (KKG-I: e.g., “If I feel not physically well, I have to attribute this to myself”), social externality (KKG-S: e.g., “If physical complaints occur, I ask an expert to help me”), and fatalistic externality (KKG-F: e.g., “I owe it to my destiny if my physical complaints disappear”). Each subscale consists of seven items that are answered on a six-point response scale from (1) “completely disagree” to (6) “completely agree.” Cronbach’s alpha for the subscales were for the KKG-I = 0.80, for KKG-S = 0.68, and for KKG-F = 0.83.

#### Coping

Coping was assessed using a five-dimensional German questionnaire (Trierer Skalen zur Krankheitbewältigung, TSK; Klauer and Filipp, 1993). The TSK consists of 37 items, which are assigned to five scales: (a) rumination (e.g., “I have brooded about whether other people are really honest and open toward me”), (b) search for affiliation (e.g., “I tried to get in touch with people who had a similar experience”), (c) threat minimization (e.g., “I told myself that I’m simply going through a bad time and can be lucky in the future again”), (d) search for information (e.g., “I tried to get information about my illness and possible treatments by talking to other people”), and (e) search for meaning in religion (e.g., “I prayed and sought comfort in my faith”). Participants were asked to refer to the period of time after the cardiac event in their answers. Items are answered on a six-point scale from (1) “never” to (6) “very frequently.” Cronbach’s alphas for the subscales were 0.74 for “rumination,” 0.87 for “seeking for social integration,” 0.79 for “threat minimization,” 0.80 for “seeking for information and sharing experiences,” and 0.82 for “searching for meaning in religion.”

### Statistical analysis

Statistical analyses comprised bivariate correlations and partial correlations on continuous data and *t*-tests for independent samples on categorical data. Because of the study’s small sample size, there was a substantial risk of Type 2 error. Therefore, the threshold for statistical significance was defined as *p* < 0.10. One-tailed tests were used for hypothesized associations, while all other tests were two-tailed.

## Results

### Preliminary analysis

In a first step, we compared patients participating in all three assessments (time 1, time 2, and time 3) with those who did not respond at time 2 (*N* = 3) and at time 3 (*N* = 12), respectively, on demographic, clinical, and psychological measures assessed at time 1 using independent *t*-tests. There were no differences between those not responding at time 2 and those participating in all three assessments. Non-responders at time 3, however, were significantly older and indicated a significantly lower perceived controllability of their illness. These differences should be kept in mind when interpreting the results including time 3.

Seven of the 36 patients (19.4%) met the DSM-IV criteria for major depression. To confirm the depression diagnoses, a *t*-test was conducted with depression as the independent factor (1 = no depression/2 = depression) and the ADS scores (time 1) as dependent variable. Patients diagnosed as depressed according to DSM-IV criteria had significantly higher ADS scores (*M* = 18.39, SD = 4.91) than patients without such a diagnoses [*M* = 9.64, SD = 6.56; *t*_(29)_ = 3.26; *p* < 0.01]. Three patients (8.4%) met the DSM-IV criteria for major depression prior to MI. A *t*-test for independent samples showed that patients with a major depression prior to MI (*M* = 16.25, SD = 7.51) did not differ significantly in ADS scores from patients without previous depression [*M* = 11.12, SD = 7.11, *t*_(29)_ = 1.18, *p* = 0.25].

In order to examine possible effects of medical and demographic variables on threat appraisal, control beliefs, coping strategies, and depressive symptoms, Pearson correlations and *t*-tests were computed on continuous data and categorical data, respectively. There were no significant associations between the clinical indices obtained at time 1 (blood lipids, number of main coronary arteries with >70% stenosis, maximum stenosis, etc.), and any of the other variables. Gender, however, was significantly related to depression scores at time 1. Women had significantly higher depression scores (*M* = 18.25, SD = 9.88) than men [*M* = 10.48, SD = 7.21, *t*_(34)_ = −2.36, *p* < 0.05]. Age showed a positive association with fatalistic externality in the KKG (*r* = 0.49, *p* < 0.01) and the coping strategy “rumination” (*r* = 0.37, *p* < 0.05). As no further systematic associations between medical and/or demographic variables on the one hand, and psychological factors on the other hand were observed, they were excluded from the final set of analyses.

As can be seen from Table 1, threat appraisal showed a significant inverse relationship with the coping strategies “search for affiliation” and “threat minimization,” while internality was positively correlated with social externality and the coping strategy “search for information.” Unexpectedly, no association could be found between threat appraisal and the coping strategy “rumination.”

**Table 1:** **Means, SD of predictor variables, and intercorrelations**.

	*M*	SD	1	2	3	4	5	6	7	8	9
Threat appraisal	54.34	14.84		0.00	0.27*	0.14	0.05	−0.32*	−0.27*	−0.08	−0.12
Internality	27.72	6.04			0.28*	0.23	0.12	0.02	0.10	0.38**	0.00
Social externality	25.45	5.23				0.17	0.26	0.01	−0.20	0.09	0.14
Fatalistic externality	22.06	7.15					0.18	0.07	−0.15	0.02	0.24
Rumination	28.53	7.43						0.46***	0.30*	0.69***	0.49***
Search for affiliation	32.58	9.23							0.57***	0.40**	0.60***
Threat minimization	39.57	4.90								0.39**	0.39**
Search for information	28.11	7.36									0.26
Search for meaning in religion	8.46	4.25									

*p < 0.10, **p < 0.05, and ***p < 0.01.

### Associations between independent and dependent variables

Bivariate correlations were calculated to examine the extent to which predictors and depression scores were related cross-sectionally and longitudinally. Five participants were excluded from this analysis as their respective dissimulation scores in the ADS exceeded the recommended maximum. Correlation coefficients are summarized in Table 2.

**Table 2:** **Correlations between predictor variables and depression at time 1, time 2, and time 3**.

Predictor variables (time 1)	Depression scores (ADS)			DSM-IV diagnosis of depression^3,4^
	Time 1^1^	Time 2^1^	Time 3^2^	Time 1
Threat appraisal	0.43***	0.38**	0.22	0.17
Internality	−0.10	−0.43***	−0.30*	−0.13
Social externality	0.02	−0.30*	0.08	−0.04
Fatalistic externality	0.20	−0.08	−0.13	−0.12
Rumination	−0.09	0.01	0.14	−0.21
Search for affiliation	−0.42**	−0.28	−0.30*	−0.26
Threat minimization	−0.36**	−0.31*	−0.19	−0.10
Search for information	−0.25	−0.13	−0.08	−0.13
Search for meaning in religion	−0.07	−0.12	−0.06	0.00

*p < 0.10, **p < 0.05, and ***p < 0.01; ^1^*N* = 31; ^2^*N* = 24; ^3^pointbiserial correlations; ^4^1 = no depression diagnoses 2 = depression diagnoses.

As can be seen, not all correlations are consistent with the hypotheses. Only higher levels of threat appraisal at time 1 were significantly correlated with higher depression scores at time 1 and time 2. Threat appraisal did not relate to DSM-IV depression diagnosis or to depression scores at time 3. There are no significant correlations between control beliefs and depression scores at time 1, but internality and social externality were significantly inversely correlated to depression scores at time 2. Furthermore, internality was significantly inversely correlated to depression scores at time 3. The coping strategy “search for affiliation” was significantly correlated to lower depression scores at time 1 and time 3, while the coping strategy “threat minimization” was significantly related to lower depressions scores at time 1 and time 2. Unexpectedly, fatalistic externality and the coping strategy “rumination” were unrelated to depression scores at all assessment points. In addition, partial correlations between predictor variables and depressions scores at time 2 and time 3 were calculated by controlling for depression scores at time 1 (see Table 3).

**Table 3:** **Partial correlations between threat appraisal, locus of control and coping strategies, and depression scores at time 2 and time 3 by controlling for depression scores at time 1**.

Predictor variables (time 1)	Depression scores (ADS)	
	Time 2^1^	Time 3^2^
Threat appraisal	0.18	−0.01
Internality	−0.51***	−0.20
Social externality	−0.40**	0.04
Fatalistic externality	−0.13	−0.29
Rumination	0.10	0.24
Search for affiliation	0.03	−0.09
Threat minimization	−0.18	−0.16
Search for information	0.12	0.26
Search for meaning in religion	−0.10	−0.15

**p < 0.05, and ***p < 0.01; ^1^*N* = 22; ^2^*N* = 17.

Only the correlations between internality, social externality, and depression scores at time 2 are significant, none of the other correlations reached statistical significance after controlling for depression scores at time 1. The main reason for this pattern of results is the high stability of depression scores over time. The bivariate correlations between ADS scores from time 1 and time 2 (*r* = 0.71, *p* < 0.01) and time 2 and time 3 (*r* = 0.69, *p* < 0.01) were highly significant. Nevertheless, cardiac threat appraisal has a small effect on depression scores at time 2 (*r* = 0.18). At time 3 no effect of threat appraisal on depression scores occurred.

It is conceivable that the categories of perceived threat as assessed by the CTQ show differential patterns of association with depression; the total CTQ score may, therefore, not be appropriate to detect any such significant relationships. Additional analyses were, therefore, carried out by correlating the subscales of the CTQ at time 1 and depression scores and depression diagnoses at time 1, time 2, and time 3.

As can be seen from Table 4, the CTQ-subscales “fatigue,” “general health,” and “disease-specific symptoms” were significantly correlated with depression scores at time 1 and time 2, while the subscales “work” and “family” were unrelated with depression scores and DSM-IV diagnoses at both times. Only the subscale “general health” was significantly related to DSM-IV depression diagnosis. In addition, the subscales “fatigue” and “disease-specific symptoms” were significantly correlated with depression scores at time 3. These results indicate that “fatigue,” “general health,” and “disease-specific symptoms” are the most relevant stressors for post-MI patients.

**Table 4:** **Correlations between subscales of the CTQ and depression at time 1, time 2, and time 3**.

CTQ-subscales	Depressions scores (ADS)			DSM-IV diagnosis of depression^3,4^
	Time 1^1^	Time 2^1^	Time 3^2^	Time 1
Fatigue	0.41***	0.36**	0.37**	0.17
General health	0.48***	0.42***	0.21	0.31**
Disease-specific symptoms	0.37**	0.36**	0.31*	−0.05
Work	0.12	0.00	0.05	0.00
Family	0.08	0.14	−0.10	0.01

*p < 0.10, **p < 0.05, and ***p < 0.01; ^1^*N* = 31; ^2^*N* = 24; ^3^pointbiserial correlations; ^4^1 = no depression diagnoses 2 = depression diagnoses.

## Discussion

The results of the present study suggest that cardiac threat appraisal and depressive symptoms in the immediate post-MI period are significantly associated. As predicted, higher threat appraisal was related to higher depression scores at time 1. In addition, the findings indicate that “fatigue,” “general health,” and “disease-specific symptoms” are the most relevant stressors for patients immediately after the MI.

In contrast to the results obtained at time 1, cardiac threat appraisal did not contribute to depression scores at time 2 (after controlling for time 1 depression scores) and time 3. It is plausible that any potential association between cardiac threat appraisal and time 2 and time 3 depression scores may have been obscured by the high stability of depression scores. An alternative explanation may be that threat appraisal changed after time 1. Unfortunately, this was not directly tested in the current study. However, anecdotal reports suggest that the appraisal measure may have assessed threat associated with current MI problems rather than with MI in general. This interpretation is supported by the fact that only the CTQ-subscales “fatigue,” “general health,” and “disease-specific symptoms” were associated with depression scores at time 1. These three subscales assess acute symptoms of the MI, while the subscales “work” and “family” concern threatening consequences, which are probably more salient when the patients are back at home. At time 1, all patients stayed at the hospital while at time 2 and time 3 they were back at home. We would argue that patients at time 2 and time 3 had other concerns (e.g., work and family) than at time 1. The return to home at time 2 might also explain the significant inverse relationship between “social externality” and depression at this measurement point, as high scores on “social externality” might reflect increased levels of perceived control in the home environment, which in turn may protect against depression. Nevertheless, the change of threat appraisal through reassessment of the situation or the stressor (reappraisal) should be clarified in further studies.

The present results indicate that patients with high scores on the CTQ use less adaptive coping styles than patients with lower perceptions of threat. There was a significant inverse association between the CTQ and the subscales “search for affiliation” and “threat minimization” of the coping questionnaire. “Search for affiliation” correlated negatively with depression scores at time 1 and time 3 while “threat minimization” correlated negatively with depression scores at time 1 and time 2. Neither the expected relationship between threat appraisal and the coping strategy “rumination” nor the hypothesized positive relationship between “rumination” and depression scores was found. It could be speculated that in the immediate time period following an MI, “rumination” *per se* might be not as maladaptive as expected (Watkins, 2008). A more detailed assessment of rumination content and style, e.g., abstract versus concrete processing of thoughts, appears promising for future studies (Watkins, 2008).

As predicted, internality is important for illness adaptation in the long run. Patients with higher internality scores had lower depression scores at time 2 and time 3, although the partial correlation did not reach statistical significance at time 3. This is in line with previous reports showing that high internality scores contribute significantly to earlier return to work in coronary patients (Bergvik et al., 2012).

Unexpectedly, fatalistic externality did not relate to higher depression scores at either point of assessment. It is possible that control beliefs changed over the period of observation, although – in the absence of any repeated assessments of control beliefs – this remains speculative at this point in time, and should, therefore, be investigated in future studies.

The analysis of the associations between coping strategies and depression scores showed a beneficial effects of the coping strategy “search for affiliation” at time 1 and time 3 in that patients who tend to draw away their attention from the MI (predominately by the involvement of other persons) felt less threatened by the MI and reported lower depression scores at time 1 and time 3. This coping strategy can be interpreted as seeking social support, and has been found to have positive effects on illness adaptation (Frasure-Smith et al., 2000). The coping strategy “threat minimization” was related to lower depression scores at time 1 and time 2. Patients using this coping strategy tend to think positive and to rationalize and minimize the cardiac event. On the other hand, the results from the partial correlation do not support the assumption that coping strategies are associated with depression scores over time.

The prevalence rate of depression after first MI observed in this study corresponds with those reported for MI patients elsewhere (Thombs et al., 2006) and is clearly higher than the estimated prevalence rate of depressive disorders in the general population (Kessler et al., 2003). Consistent with previous research (Zahn et al., 2010) clinical indices of disease severity were not related to depression scores and only gender as a patient demographic characteristic was associated with depression scores. In general this finding underlines the role of psychological factors for post-MI depression.

### Limitations

The present study is limited by a non-random sample, a relatively small sample size, and a moderately high drop-out rate at time 3 which limits the generalizability of the present findings. Secondly, causal directions remain ambiguous because most of the associations are derived from cross-sectional analyses, and because of the correlational design. At time 2 and time 3, only measures of depression were obtained while the other variables obtained at time 1 were not included. In order to replicate the present findings and to analyze the adaptation process more thoroughly, all independent and dependent variables should be assessed at all points of measurement in future studies. Furthermore, larger sample sizes are desirable in order to make use of structural equation modeling and cross-lagged panel analysis.

## Conclusion

Despite the limitations of the present study, some practical implications can be derived from these findings. Firstly, our findings suggest that psychological interventions aimed at reducing cardiac threat appraisal over a period of at least 2 weeks after the acute MI could contribute to lower depression and thereby increase the prospect of a smooth recovery after MI (Cossette et al., 2001). Cognitive interventions including specific information about the illness and allowing the patient re-interpretation of his/her situation would appear to be best suited to minimize the threat potential of MI. Furthermore, stress management training might be applied through teaching problem-solving strategies and ways to reduce reliance on passive coping styles. Patients at risk should be identified as early as possible and receive appropriate treatments. These interventions might help to optimize the stress-coping process. Further studies are needed to investigate the effects of such treatments.

## Conflict of Interest Statement

The authors declare that the research was conducted in the absence of any commercial or financial relationships that could be construed as a potential conflict of interest.

## Abbreviations

MI, myocardial infarction.
